# Editorial: Clinical risk assessment and intervention of gastrointestinal tumors driven by big-data

**DOI:** 10.3389/fmed.2024.1379762

**Published:** 2024-02-27

**Authors:** Nan Zhang, Wei Wang, Xin Gao, Feng Gao

**Affiliations:** ^1^Department of Gastroenterology, The First Hospital of Jilin University, Changchun, China; ^2^Department of Pathology, First Affiliated Hospital of Anhui Medical University, Hefei, China; ^3^Computational Bioscience Research Center, King Abdullah University of Science and Technology (KAUST), Thuwal, Saudi Arabia; ^4^Computer Science Program, Electrical and Mathematical Sciences and Engineering Division, King Abdullah University of Science and Technology (KAUST), Thuwal, Saudi Arabia; ^5^Department of General Surgery, Department of Colorectal Surgery, The Sixth Affiliated Hospital, Sun Yat-sen University, Guangzhou, China; ^6^Guangdong Provincial Key Laboratory of Colorectal and Pelvic Floor Diseases, The Sixth Affiliated Hospital, Sun Yat-sen University, Guangzhou, China; ^7^Biomedical Innovation Center, The Sixth Affiliated Hospital, Sun Yat-sen University, Guangzhou, China; ^8^Shanghai Artificial Intelligence Laboratory, Shanghai, China

**Keywords:** individualized medicine, medical knowledge, biomedical data analysis, tumor microenvironment, prognosis

The molecular mechanisms underlying tumor development are highly intricate. For gastrointestinal tumors, comprehending and controlling the heterogeneous molecular mechanisms may unveil novel therapeutic targets (1, 2). In recent years, the cancer field, including gastrointestinal tumors, has generated an abundance of molecular and phenotypic data. Facilitated by high-throughput technologies, a vast amount of genomic data has rapidly accumulated, giving rise to the era of cancer “big data.” These cancer datasets continue to expand globally, fueled by investments in the research community (3). The utilization and development of extensive databases play a pivotal role in establishing a sturdy framework for deciphering the molecular mechanisms involved in tumor initiation and progression, while concurrently exploring innovative approaches to diagnosis and treatment. Within this context, it is possible to achieve precise assessment of clinical risks using bioinformatics and identify suitable biomarkers through multi-omics data. This enables personalized and precise diagnosis and treatment for patients with gastrointestinal tumors. The present study compiles some of the outcomes in these areas, offering new insights and perspectives for the diagnosis, prognosis, and individualized treatment of gastrointestinal tumors.

Inherent heterogeneity characterizes gastrointestinal tumors (4), and this diversity extends to the tumor microenvironment (TME), exerting a significant influence on disease progression and treatment response (5). In a study by Xu et al. conducted a study on Helicobacter pylori-related genes (HRGs) expression patterns in gastric cancer. They identified two prognostically distinct subtypes and developed a Cancer-Associated Fibroblast Differential Expression Gene scoring model. This model, a first of its kind, predicts the prognosis of *H. pylori*-associated gastric cancer patients, guiding precise treatment. The study reveals *H. pylori's* impact on tumor-associated fibroblasts (CAFs), influencing the tumor microenvironment and disease progression. Concentrating on HRGs, researchers unveiled heterogeneity, with subsequent analyses emphasizing the crucial role of CAFs and providing novel insights into malignant progression mechanisms. In a separate study, Shi et al. developed a deep learning model employing the VGG19 structure and a transfer learning strategy. This model integrates TME features and clinical variables for prognosis prediction. By uncovering key pathways related to TME signals, the biological basis of TME signal prediction for prognosis in colorectal cancer (CRC) patients is explored. This contributes to the significance of prognosis for CRC patients, aiding in their treatment and management.

In recent years, there has been a notable increase in the incidence of early-onset colorectal cancer (EO-CRC), diagnosed before the age of 50 (6). Researchers are increasingly focused on the prevention of colorectal cancer. Song et al. in their evidence-based medicine research, propose that persistent obesity and continuous abdominal obesity before the age of 50 are linked to an elevated risk of EO-CRC. An effective strategy for colorectal cancer prevention involves the utilization of colonoscopy (7). However, incomplete polyp removal can lead to 10%−28% of interval colorectal cancers (cancers discovered after screening or monitoring colonoscopy) (8). Li et al. through evidence-based medicine research, found that for polyps with a diameter of 4–10 mm, endoscopic mucosal resection (EMR) and hot snare polypectomy (HSP) are superior to cold snare polypectomy (CSP) in improving the complete removal rate. These methods can be relatively safely applied to patients not taking antithrombotic drugs. Additionally, Chen et al. in a meta-analysis, have identified lactic dehydrogenase (LDH) and NLR-LDH as dependable, straightforward, and independent biomarkers for predicting synchronous or metachronous liver metastasis of early colorectal cancer (CRLM) and overall survival (OS) in colorectal cancer patients. The neutrophil-to-lymphocyte ratio (NLR) stands out as a crucial monitoring indicator for CRLM patients, contributing to the enhancement of surgical resection rates and the extension of survival.

In summary, research on gastrointestinal tumors based on big data has made remarkable strides in personalized medicine. This study has aggregated scientific research articles focusing on the risk assessment and intervention strategies related to gastrointestinal tumors. These findings have not only advanced our conceptual understanding of cancer biology but are also poised to influence diagnostic and treatment decisions for gastrointestinal tumors, contributing to clinical practice.

## Author contributions

NZ: Writing – original draft, Writing – review & editing. WW: Writing – original draft. XG: Writing – review & editing. FG: Writing – review & editing.
